# An Energy-Saving Forwarding Mechanism Based on Clustering for Opportunistic Networks

**DOI:** 10.3390/s21227427

**Published:** 2021-11-09

**Authors:** Gilmara Santos, Diogo Soares, Celso Carvalho, Edjair Mota

**Affiliations:** 1 Institute of Computing, Federal University of Amazonas, Manaus 69080-900, Brazil; diogo.soares@icomp.ufam.edu.br (D.S.); edjair@icomp.ufam.edu.br (E.M.); 2 Postgraduate Program in Electrical Engineering, Federal University of Amazonas, Manaus 69080-900, Brazil; ccarvalho_@ufam.edu.br

**Keywords:** opportunistic networks, energy-saving, clustering

## Abstract

In Opportunistic Networks (OppNets), mobility of and contact between nodes are explored to create communication opportunities and exchange messages and information. A basic premise for a better performance of these networks is a collaboration of the nodes during communication. However, due to energy restriction factors, nodes may eventually fail to collaborate with message exchanges. In this work, we propose a routing mechanism called *e*GPDMI to improve message probability of delivery while optimizing nodes’ energy consumption. Unlike other algorithms proposed in OppNets literature, *e*GPDMI groups nodes by energy level and nodes interests using clustering techniques. Our major assumption is that retaining messages in nodes with the highest energy levels can improve network performance, thus overcoming the problem of nodes’ disconnection due to unwillingness to cooperate due to low energy values. Through questionnaire application and factorial design experiments, we characterize the impacts of energy levels in OppNets. Further, we apply performance evaluation of the *e*GPDMI mechanism in terms of effectiveness using mobility from real-world scenarios. The results show that our mechanism effectively reduces the degradation of the probability of delivery when the minimum energy level used for nodes to cooperate with communication increases.

## 1. Introduction

Recent advances in wireless networks have boosted promising paradigms such as communication between people directly from their own devices without using network infrastructure. The global Internet traffic reaches higher values year after year, mainly because of (i) the increasing number of personal devices, such as smartphones, tablets, or wearable devices; (ii) the diffusion of content-oriented services, such as chats, streaming, and content shared among users. It has become a considerable challenge for Internet providers to meet this constantly higher traffic.

Some paradigms of infrastructure-less communications emerged to address these situations, such as the Opportunistic Networks (OppNets). OppNets are a particular case of delay-tolerant networks (DTN), where messages exchange occurs during contact opportunities when mobile nodes are in the same coverage area.

Because mobile devices use resources to share data, OppNets can overcharge resources such as device energy or device buffer. Buffer management for mobile devices still draws the attention of researchers [1]. However, we found a lack of in-depth studies related to energy level or consumption and their impact on OppNets [2,3].

Therefore, one should consider energy consumption as a relevant metric for performance evaluation [4]. In [5], the authors claim that the communication process is mainly responsible for the energy consumption of devices in wireless communications. Energy usage can have different effects on OppNets’ performance evaluation. While mobile devices with a fully discharged battery cannot take part in data transmission, those with low battery levels may not be encouraged to cooperate on opportunistic communications [6,7]. It is well known that data transfer and the discovery of nearby devices consume energy. In addition, after turning a device off, it is not possible to predict when it will be available again, affecting the routing success.

The battery usage pattern depends on the user’s behavior. The work of Bulut and Dhungana [8] examines the problem of energy balance in OppNets from the social structure of the network, considering energy balance with social awareness as an important strategy. We conceived, designed, and implemented a routing protocol that groups nodes according to their battery usage pattern, classifying them by high and low power levels, with forwarding decisions based on devices with similar power levels, considering the high levels.

The proposed mechanism (*e*GPDMI) extends the GPDMI opportunistic routing protocol by Neves et al. [9], taking advantage of its essential characteristics of clustering the nodes around their interests and social-based mobility. That assumption relies on the fact that, in OppNets, nodes tend to cooperate in communication if message content is in their interest [10].

From the OppNets perspective, these network characteristics are attractive to share interests using mobile devices contacts. This article assumes that the message dissemination considers the interests of the users carrying the mobile devices. Therefore, the proposed mechanism intends to apply a forwarding decision based on the nodes’ energy consumption, combining the nodes’ social interests over the nodes.

The idealized routing mechanism creates groups of nodes according to both the energy levels and the similarity of social interests. This combination avoids trying to forward messages through nodes that are considered selfish because of the limited available energy. We present the motivation for this investigation and compare its performance against other routing mechanisms in the literature.

This paper is organized as follows. Section 2 presents some related works. Section 3 shows our study on energy effects over opportunistic networks. Section 4 describes the architecture overview of the proposed mechanism. Section 5 describes the deployed methodology. Section 6 depicts the performance evaluation results. Finally, Section 7 presents our conclusions and future works.

## 2. Related Works

Recent forwarding strategies adopt social aspects at the core of their decisions. OppNet users carry mobile devices; thus, it is reasonable to claim that social network models are suitable for designing routing protocols [11]. Most of them emphasize the role of social aspects in the design phase, such as tie strength, popularity, or centrality metrics [12,13].

Recently, Ying et al. [14] researched opportunistic routing algorithms that use social features to stimulate the performance of message forwarding. The authors conducted comparative research on these algorithms, analyzing three social characteristics, namely centrality, similarity, and tie strength, in addition to simulation experiments, demonstrating the impact of these algorithms on the network. They reinforced the idea that, although much work has already been performed on opportunistic routing algorithms, they still need to explore social features more effectively in designing new routing algorithms.

Despite the significance of social aspects, resource constraints also play an essential role in nodes deciding to cooperate in network transactions. Whereas opportunistic contacts are the primary reason to yield communication, the node’s unwillingness to cooperate affects the network performance and may be harmful [15]. In a questionnaire applied by Souza et al. [16], 10% of the mobile users at the Federal University of Amazonas (UFAM) claimed they would share data with other devices, even though they had a low battery level, and 55% of the users would prefer to recharge their battery fully. Gupta et al. [17] showed that network performance degrades to about 62% when the proportion of non-cooperating nodes goes from 0% to 50%.

In OppNets, nearby devices may cooperate by sharing data. However, the interaction and data transfer among nodes can consume different energy levels depending on the operation, since mobile nodes are battery operated. Therefore, even discovering if a neighbor device is near may consume an extensive amount of energy. In [17], the authors define the following modes as energy-consuming: scanning, transmission, reception, and inactive mode.

A study by Loudari and Benamar [18] on the evaluation and comparison of energy consumption in OppNets takes into account mobility models relating them to energy consumption and selfish nodes, concluding that routing protocols in opportunistic networks focus on decreasing the average delay and the overhead and increasing the message delivery rate. For this, they increase the number of copies of packets on the network. Therefore, more energy is consumed by the nodes, making the environment more hostile, as the energy consumption plays a crucial role in modifying the behavior of nodes in the network because nodes with low battery power can become selfish and uncooperative.

The authors Yu et al. [19] emphasized the formation of communities based on social characteristics, proposing an opportunistic network routing strategy based on individual communities of nodes. The algorithm builds social relationships through the probabilistic encounter between them; individual communities are formed based on the centrality of nodes.

Another approach in [20] is to form user clusters through the probability of meeting between the nodes. This reflects the degree of connection between them, which can be used for forwarding future messages. The idea is that users with a high probability of finding each other must be in the same cluster.

The work of Hui et al. [12] demonstrates that social communities positively affect data transmission. However, in [2], the authors conclude that using social characteristics might be unfair and may cause node overload. Their results showed that only 10% of nodes handle 63% of all data traffic. Junior and Campos [3] proposed a modified version of Bubble Rap [12] by randomly choosing some nodes among the most central (a popularity metric) nodes to reduce their load.

An approach used in [21] proposes a protocol based on energy-efficient inactive nodes detection. It is a method to detect nodes in a dead or inactive state in the network and provide an efficient buffer management policy to avoid network congestion. In summary, there is a focus on controlling the transmission of messages destined for dead nodes, eliminating messages destined for them and thus avoiding the consumption of nodes resources.

In line with the authors mentioned above, Neves et al. [9] demonstrated clustering methods to improve delivery rates in scenarios with social aspects and message interest profiles. They applied the clustering algorithms EM [22] and k-means [23] to build groups with common interests. They compared the performance of their proposed method (GPDMI) with Bubble Rap [12], which also uses clustering techniques to create social communities.

Their results showed that, by introducing interest profiles and social aspects in the OppNet context, clustering nodes by interest profiles is the most suitable choice to improve the dissemination of messages.

Since there are more interactions among the nodes with over one common interest topic, message forwarding from the source to the cluster of the topic of interest occurs over the nodes’ path with an indirect interest in the message. Although GPDMI performs better than Bubble Rap, we realized a gap in performance evaluation when considering energy constraints. It is essential to emphasize that, though our approach is similar to GPDMI, we added a new clustering layer above to group the nodes with the highest battery level.

Our mechanism considers the relationship of users’ mobile device usage with battery consumption. The idea is to group them by energy levels, assuming that there is a user behavior correlation to the daily use of their mobile device so that this history can be exploited to optimize the energy resources of the nodes when forwarding messages on the network.

Amah et al. [24] investigated the burden and fairness of forwarding strategies in OppNets. Using mobility traces, they found it difficult to estimate the node’s burden determinant factors. The authors claim that resource usage depends on variables unrelated to nodes’ contribution in forwarding messages, such as TTL, number of contacts, queuing policy, and routing protocol.

## 3. Energy Impact

The battery level is the remaining energy in the user’s device used by the user’s applications and communication among nearby devices. However, when this resource is low, we assume the nodes will not cooperate in opportunistic communication. Network metrics can degrade if a node leaves the network operations, as fewer nodes store the messages. To test this hypothesis, we carried out a questionnaire to evaluate two issues:

(i). the behavior of real users when taking part in OppNet at a critical energy level,
(ii). from which level of energy users consider taking part in the network only favoring themselves (not accepting messages from other users).

We conducted this experiment on the UFAM campus Manaus with 351 participants of several courses to answer these questions. The research questions and alternatives can be seen in Table 1.

The questionnaire result showed that 77.16% of the users would not accept the request to take part or wait for their batteries to recharge. This result corroborates the null hypothesis that nodes may leave network operations of storing data to forward later. One can analyze the cooperation behavior by using the second question as a guide.

We found that 68.10% of the users would no longer cooperate in communication if the battery was lower than 30%. Only 19.65% of the users agreed to cooperate with the network operation if their energy level was above 50%.

This result reinforces the hypothesis that low energy nodes would not cooperate with the network forwarding mechanism.

We applied another experiment that highlights the impact of cooperation based on the energy level. This analysis can allow us to choose an appropriate design of experiments; it also defines an adequate set of investigation factors.

### Factorial Design

A performance evaluation must comprise two key components, the design of experiments, which refers to planning the experiments to collect the data feasibly for statistical analysis, and the actual statistical analysis of the data [25]. We applied a 2k factorial design, a technique comprising two or more factors as input, representing some of the network parameters that significantly influence the performance metrics.

Factorial design helps sort out the factors according to their impact and points out how their interactions can impact performance metrics. The basic approach for factorial design is to select a set of k factors with two levels (represented as −1 and 1). We simulated all combinations of k factors with 2^k^ experiments. Since we were interested in the impact of energy level, we defined the factors in Table 2. Our response variable *y* is the probability of delivery of the messages. The coded variables were used in a regression non-linear model built to represent the effects of the factors and all their possible interactions on the variable, given by Equation (1). It is important to highlight that we carry out the factorial design without replication.

*y* = *q*_0_ + *q*_A_*^x^*_A_ + *q*_B_*^x^*_B_ +*q*_C_*^x^*_C_ + *q*_D_*^x^*_D_+ *q*_AB_*^x^*_A_*^x^*_B_ + *q*_AC_*^x^*_A_*^x^*_C_ +…+ *q*_BC_*^x^*_B_*^x^*_C_ +…+ *q*_ABCD_*^x^*_A_*^x^*_B_*^x^*_C_*^x^*_D_ (1)

In order to find out the effect of each factor (explained in Table 3) on the response of interest y, one can assign minimum and maximum levels to the selected factors (see Jain’s book [26] for more details) and record the result of the probability of delivery of the message obtained by simulation.

We determined the density of the scenario as a factor to analyze how scenarios can affect the simulation results. We defined two battery ranges, so the values represent which battery level node keeps cooperating in the network and carrying messages from other nodes. In the experiment’s execution, we used an epidemic routing algorithm. In the minimum value (between 75% and 100%), nodes cooperate only if their battery levels are in this range (low cooperation). In the maximum value of (0% the 100%), nodes carry messages from other nodes regardless of their remaining energy level (high cooperation).

Since the scenario needs to be similar to the real-world ones, we chose a sparse (Reality) and a dense (Infocom), extracting information from real experiments. In Section 5.1, we present further details for the selected scenarios.

Table 3 presents the investigation of the effect of the node’s battery charge level on the message’s probability of delivery and the levels attributed to each factor in the simulation. The minimum and maximum levels are assigned to variables of set X = {x1, x2, x3, x4}, which alternate their levels according to Table 3. For example, x2 = −1 is assigned to battery range = [75, 100] and x2 = 1 is assigned to battery range = [0, 100].

The next step is to calculate the percentage of message delivery variance attributed to each of the four factors and each interaction between them. The percentage of variation captures the importance of each factor in the response variable.

Table 4 describes the percentage of variation pointed out by the factorial design. The battery range factor has a more significant impact on the message’s probability of delivery (76.96%). The scenario and the joining factor composed of both battery range and scenario explain 18% and 3% of the variation. Thus, the remaining node energy necessary to cooperate is the most significant factor that deserves further analysis.

Although the impact of cooperation has been studied previously [17], to the best of our knowledge, this is the first study that applied factorial design to evaluate the impact of energy level on the messages delivery probability compared to other OppNets parameters.

Further, we highlight scenarios that also have a relevant effect on the performance metric evaluated.

**Figure 1:**
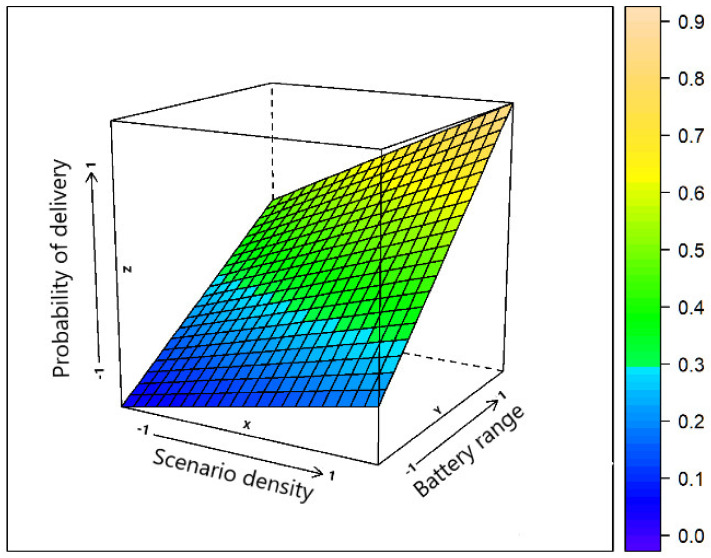
Impact of the scenario density and battery range on the delivery probability of messages.

Figure 1 presents the correlation between the battery range and the density of the scenario, both related to the result of the message delivery probability metric.

When the density of the scenario is higher (more contacts) and the battery level is in the range *E* = [0, 100], which means full cooperation of the nodes in the network, message delivery probability is higher than in the sparse scenario or if the range battery is out of range *E* = [75, 100].

## 4. Architecture Overview

In this section, we describe the assumptions used in the simulations and the proposed mechanism.

### 4.1. Network Model

An opportunistic network can be modeled as a graph G (*N*, *E*), comprising a set of N vertices representing the nodes and a set of E edges representing the contacts between the nodes. Initially, each node has an initial random energy value. The energy level decreases when one of the following operations occurs: neighbor scanning, message transmission, or reception.

We assumed the nodes did not recharge their batteries during the simulation. After the full discharge of the node battery, they did not take part in the network operations. The mobility relied on human interactions by using contact traces extracted from real experiments.

### 4.2. Interest Profiles

Message interest is represented by a finite set M of distinct parts (A.K.A. “topics”). At the start, each node has a subset M of messages of interest. Two nodes (i, j) do not have common interests if Mi ∩ Mj = Ø. So, we assumed that nodes interests are static and do not change during the simulation run. Each message assigned to node “i” is initialized with one interest, randomly chosen from Mi.

### 4.3. Detailed Design

Our mechanism comprises nodes with the highest energy level values to forward messages and avoid network resource loss. Additionally, *e*GPDMI focused on situations of gathering nodes by similar interests. We applied two clustering phases to enable decision-making:

(i). the first phase clusters the nodes by energy level;
(ii). the second phase clusters the nodes according to the interest profile.

For building clusters, we analyzed two well-known clustering methods:

- EM (expect-maximization) is a clustering technique based on a statistics model called “finite mixtures”. A finite mixture is a set of k distribution probabilities representing k clusters and the values that define each cluster. EM is a method to find the maximum likelihood for a group of parameters:

1. Step 1 (expect step): It calculates the likelihood of the dataset by associating each object *x_i_* to the cluster *C_k_* by applying Equation (2).

(2) $$P\left(x_{i}\in C_{k}\right)=p\frac{C_{k}}{x_{i}}=\frac{p\left(C_{k}\right)p\left(x_{i}/C_{k}\right)}{p\left(x_{i}\right)}$$

2. Step 2 (maximization step): Applying the maximization of Equation (1), recalculating the unknown values.

(3) $$mk=\frac{1}{n}\overset{n}{\underset{i=1}{\sum }}\frac{x_{i}p\left(x_{i}\in C_{k}\right)}{\Sigma_{j}p\left(x_{j}\in C_{j}\right)}$$

EM repeats the steps above, and the algorithm ends when the maximum likelihood criteria are satisfied.

- K-means: Unlike hierarchical clustering, where the clusters merge or partition at each iteration, k-means partitions the clustering into precise k groups. The aim is to find clusters as homogeneous as possible. Hence, k-means can be written as follows.

1. Each cluster *C_k_* is randomly built by applying a random centroid *α_k_k* for each cluster;
2. For each input y, assign it to the cluster *C_k_*, which centroid *α_k_* is the closest for each input;
3. Update their centroids by using all inputs assigned for each class;
4. Calculate the error function by applying Equation (4):

(4) $$E=\overset{K}{\underset{k=1}{\sum }}\underset{y^{l}\in C_{k}}{\sum }||y^{\left(l\right)}-\alpha_{k}||{}^{2}$$

5. Repeat steps 2, 3, and 4 until there is no change in any clusters.

EM allows a more stable control over the characteristics of the clusters. Both yield a sub-optimal solution since there is a lack of global knowledge of the network status. Further, this solution is suitable for routing through groups in OppNets, where there is no central network infrastructure.

- Cluster-based forwarding: The primary goal of our mechanism is to mitigate the unnecessary use of device resources, particularly energy level, prioritizing the message transmission from nodes with a low level of energy. We assume these nodes have a stronger trend to become selfish due to a lack of resources.

Our mechanism has three main steps, (i) apply clusterization techniques to arrange neighbor nodes according to their energy levels; (ii) apply clusterization techniques to group neighbor nodes with common interests; (iii) validate the threshold of the neighbor’s energy during each contact.

The energy level threshold is defined as *E* = [Energy*lower*, Energy*upper*], *E* ⊂ [0, 100]. For example, if *E* = [50, 100], our mechanism builds a cluster with nodes with an energy level between 50% and 100%.

Depending on this condition, each node taking part in a contact forwards or does not forward a message towards a node not belonging to this cluster.

Our complete message forwarding mechanism is written as follows:

1. Message creation: Each created message is classified according to the topics of interest of the source node.
2. Classification of interests: Each node is classified according to its topics of interest.
3. Classification of energy: Each node is classified according to its energy level.
4. Clustering: The clusters of interests are built by applying the same mechanism presented in GPDMI. Then, we carry out a new clusterization run based on the energy level. We deploy the EM technique to gather nodes by energy.
5. Decision-making: The forwarding process verifies whether the node on the receiving side is in the cluster with the highest energy level and the topic of interest.

This work extends GPDMI by adding steps 3, 4, and 5. We used training data to define low energy or high energy to train the clusters by energy level, defined by *E* = [Energy*lower*, Energy*upper*]. The interests are globally represented, and every node knows the interests of all the other nodes. The node interest did not change during the simulation.

## 5. Methodology

We compared the performance of *e*GPDMI to the performance of two other strategies based on social interactions and message replications, namely PRoPHET and GPDMI. The following subsections detail the settings of this quantitative experiment.

### 5.1. Mobility Datasets

We designed a set of experiments based on stochastic simulation using the tool ONE (Opportunistic Network Environment) [27]. We selected the scenarios Reality Mining [28] and Haggle-Infocom5 [29] once they resulted from real experiments. Table 5 presents the simulation parameters.

### 5.2. Simulation Parameters

We initialized each node with a random energy level between 0 and 100%. We defined four ranges to battery capacity to use as a decision criterion during message forwarding in the mechanism presented.

We considered that, in the defined ranges, the nodes continue to cooperate with the network, forwarding the messages.

The battery capacity ranges were extended for PRoPHET and GPDMI strategies. The decision-making processes of those routings also considered the energy level. Table 6 presents detailed parameters. We repeated each simulation ten times in each set of parameters defined, and for each metric used, we computed the average values of all executions.

### 5.3. Modeling of Groups

To define the groups of nodes within the range battery capacity, we applied the intervals specified in Table 6. These values were used to build both groups of energy, the low energy level nodes and the high energy level nodes. In addition, interest groups were formed from topics obtained using the Haggle-Infocom5 mobility data set [28].

**Figure 2:**
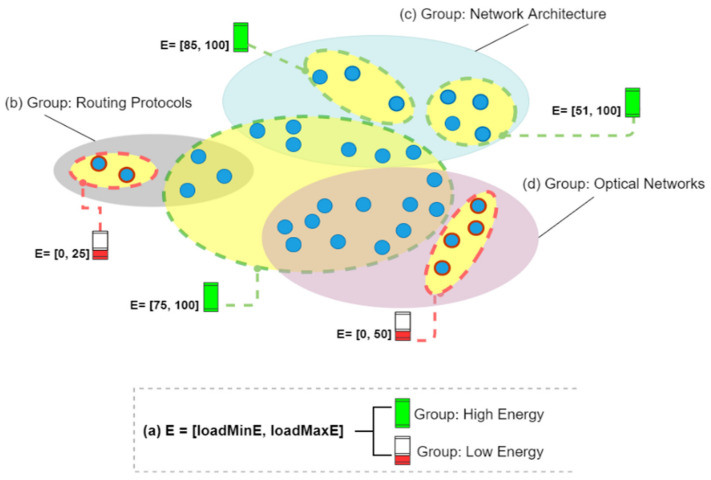
Formation of groups by energy levels and common interests.

Table 7 describes how the groups were created. We used a set of 30 different topics of interest. Then, we set *k* = 30 in the clustering technique k-means. Figure 2 is an example of group formation, where (b), (c), and (d) represent the nodes grouped according to their interests, and (a) demonstrates the use of established energy level ranges (*E* ⊂ [0, 100]) to generate the energy groups. This can be seen in the image, where we have groups of nodes with low and high battery levels. Figure 2 illustrates how clusters intersect in this strategy.

## 6. Performance Evaluation

Each simulation sequence performed the data collection to guarantee a 95% confidence interval for the estimated parameters. For each simulation run, the metric of interest was the message delivery probability, average delay, and overhead.

We applied statistical tests to estimate the efficiency of our mechanism regarding the related proposals. We divided the analysis into two subsections for each compared scenario.

### 6.1. Statistical Tests

In this paper, we applied the test “analysis of variance” (ANOVA), a statistical technique to verify potential significant difference among means and if these factors can influence the outcome variable.

ANOVA allows the comparison of several groups using continuous variables, and the means of outcome variables are computed within an acceptable margin of error. This error is referred to as α probability, and we selected the following values, 1%, 5%, or 10%.

In our performance evaluation, we used a significance level of 5%, *α* = 0.05, so we could verify the hypothesis H_0_: *µ*1 = *µ*2 = … = *µc* and H_1_, where the means are statistically different. So, the hypotheses applied in this paper were:

H_0_—Null Hypothesis, where the means do not differ.

H_1_—Alternative Hypothesis, where the means differ, i.e., at least one mean value is different from others. To evaluate the variance of an object, we applied Equation (5).

(5) $$SQTotal=\overset{I}{\underset{i=1}{\sum }}\overset{J}{\underset{j=1}{\sum }}y^{2}ij-C,\;where\;C=\frac{{\left(\sum_{i=1}^{I}\sum_{j=1}^{J}yij\right)}^{2}}{IJ}$$

Furthermore, the sum of the squares of the residuals is obtained from the difference:

*SQRes* = *SQTotal* − *SQTrt* (6)

where *SQRes*, the variance within groups, is a function of differences between the same treatment repetitions. The *SQTrt*, variation between groups is the variance between different treatments. The mean squares are calculated as follows:

(7) $$QMTreat=\frac{SQTreat}{\left(I-1\right)},\;QMRes=\frac{SQRes}{\left(I\left(J-1\right)\right)}$$

When applied to more than two treatments, we cannot designate the best treatment for the variable outcome in case of a significant difference among samples. We also applied a statistical test to complement the results found with ANOVA.

Tukey test is based on minimum significant difference (HSD), defined by Equation (8):

(8) $$\Delta =q_{k,gl,\alpha }\sqrt{\frac{QMRes}{r}},\;hsd=q\cdot \sqrt{\frac{QMRes}{r}}$$

where *q* is the total studentized amplitude given by the division of amplitude and standard deviation *s* for *k* means (number of treatments), and *gl* is the degree of freedom at the level *α* of the residue’s significance.

*QMRes* is the mean square of the residue, and *r* is the number of repetitions. If the contrast is greater than Δ (*hsd*), the means differ at the significance level or Δ < *α*.

### 6.2. Scenario Infocom

**Figure 3:**
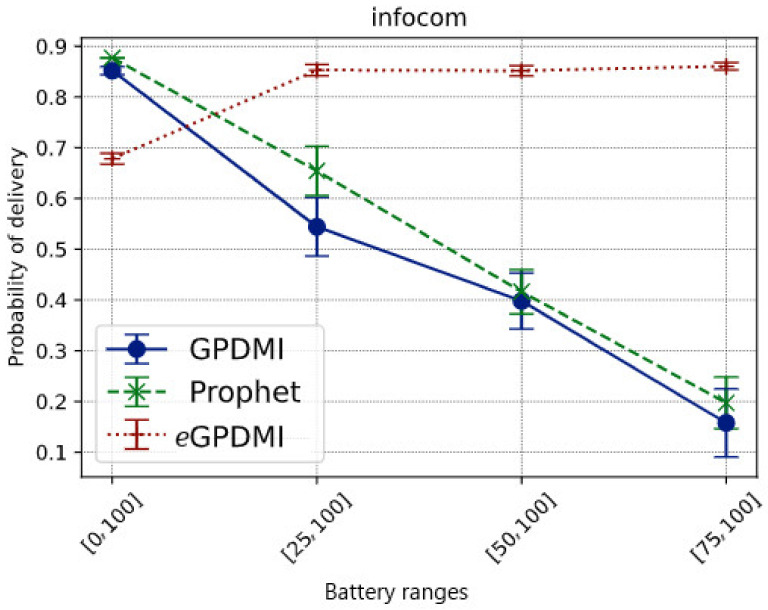
Impact of battery ranges on the delivery probability of messages in the Infocom mobility scenario.

Figure 3 shows the message delivery probability in each battery range for each tested routing algorithm. When applying the clustering layer for energy level over the other decision-making factors of the routing algorithms, we intended to keep messages alive, carried by nodes with the highest level, thus, mitigating the delivery rate degradation. The results confirm this intuition, since *e*GPDMI achieved the best message delivery probability when nodes became less cooperative to save energy.

While in the base case *E* = [0, 100], where all nodes cooperate regardless of the battery level, our approach had a message delivery rate of approximately 70%. The other routing algorithms had about an 85% delivery rate. Regarding the probability of delivery of the message of *e*GPDMI, a reasonable justification for this result relates to the fact that *e*GPDMI groups the nodes in two groups, energy levels and interests. As there was no restriction on energy level, only interests were taken into consideration. Thus, for PRoPHET and GPDMI, the flooding was higher since there was no restriction on energy. Hence, the number of replicated messages on the network was higher when compared to *e*GPDMI, as can be seen in Figure 5. Even with a smaller number of replicated messages (142.85% lower), *e*GPDMI achieved a probability of delivery only 19.35% lower than the other two protocols.

When nodes with less than 75% battery level did not cooperate (*E* = [75, 100]), *e*GPDMI had about an 80% message delivery rate, while the other routing algorithms had a delivery rate below 25%. The reasoning is that these algorithms decide to forward for nodes most likely to have new contacts, not necessarily the nodes with the highest battery levels.

**Figure 4:**
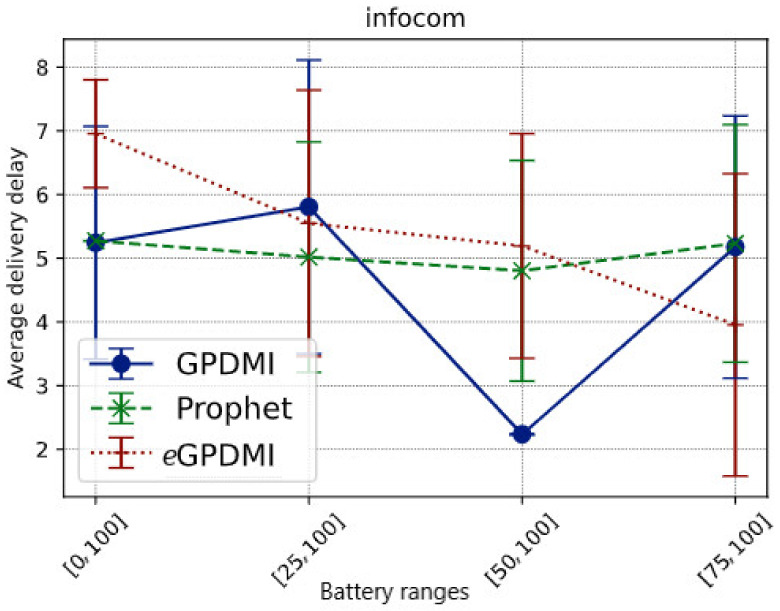
Average delivery delay vs. battery ranges in scenario Infocom.

It is important to highlight that *e*GPDMI had a better performance (approx. 104.76%) in message delivery probability when compared to GPDMI and PRoPHET. In the result of the average delay (Figure 4), *e*GPDMI performed better in case *E* = [75, 100]. Therefore, to verify if there was a significant difference between the means, we applied the *t*-test, whose result demonstrates that both *e*GPDMI and GPDMI did not present a statistically significant difference (*p*-value = 0.3904). Similarly, the results comparing *e*GPDMI and PRoPHET (*p*-value = 0.3505) did not reach sufficient conditions to reject the null hypothesis. In summary, as shown in Figure 3, the probability of delivery was higher for the case *E* = [75, 100] without substantially increasing the average delivery delay of *e*GPDMI with regard to the other routing protocols, as can be seen in Figure 4.

In order to measure overhead, we defined overhead as follows:

(9) $$\mathrm{overhead}=\frac{\left(\#\mathrm{relayed}\_\mathrm{msg}-\#\mathrm{delivered}\_\mathrm{msg}\right)}{\left(\#\mathrm{delivered}\_\mathrm{msg}\right)}$$

**Figure 5:**
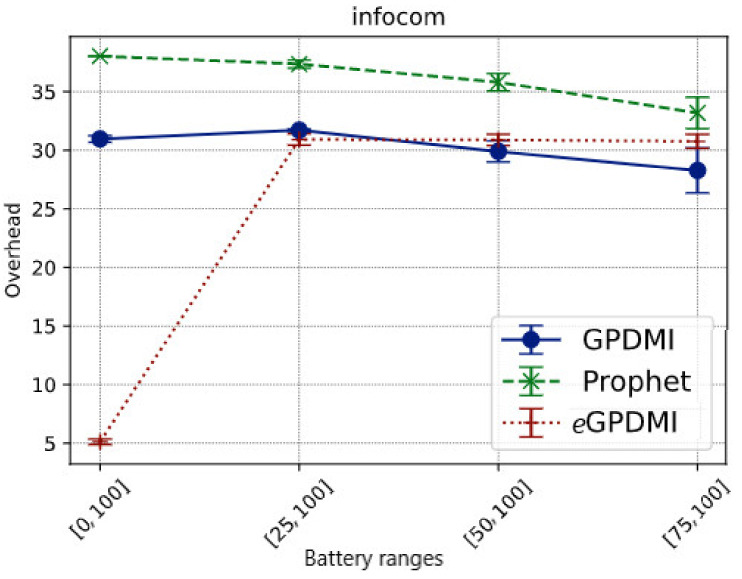
Network overhead vs. battery ranges in Infocom scenario.

As shown in Figure 5, the overhead of our proposal (*e*GPDMI) is significantly lower in case *E* = [0, 100]. The results show that messages were relayed mostly to the nodes with the highest energy level, even if the nodes cooperated with a low battery level. Further, in this scenario, *e*GPDMI forwarded fewer messages than the other algorithms.

On the other hand, when the battery load limit to forward increased, we noticed that *e*GPDMI relayed more messages than GPDMI and PRoPHET. These results imply that *e*GPDMI can keep messages alive longer than GPDMI and PRoPHET.

### 6.3. ANOVA—Delivery Probability of Messages—Infocom Scenario

When applying the ANOVA statistical test in the messages delivery probability, our results show a significant difference in the battery ranges used, as shown in Table 8. Analyzing the results of the ANOVA, it is observed by the *F* test using 5% significance that *F* > *Fcritical*, that is, there was at least one routing algorithm with a different message delivery performance between the battery range studied.

So, there is a variance in means captured by each algorithm and battery range (*F* = 409.58 and *F* = 295.17). This result supports the performance difference in delivery rates between the routing algorithms previously described in Figure 3 (delivery probability graph).

Thus, the null hypothesis H_0_ is rejected, where all averages are equal. To specifically address where this variation is occurring, we applied the Tukey test.

### 6.4. Tukey Test—Infocom Scenario

Equation (8) previously described in the subsection performance evaluation was applied to use the Tukey test in the Infocom scenario. By applying the equation to the values found in the ANOVA calculation and the other variables, we have the following result HSD = 0.058.

The HSD (minimum significant difference) is used to identify, through statistical tests, which algorithms are the same or different in the delivery performances according to the tested energy ranges. When the absolute difference of the averages (average module) calculated is greater than the value of the HSD, we say that the averages of the delivery rates of the algorithms are statistically different.

We can conclude from the results that there are relevant differences in the average performance of the delivery rates between all routing algorithms for the case where the battery range is *E* = [25, 100].

*e*GPDMI maintained the highest results with an approximately 80% delivery rate, compared with PRoPHET and GPDMI. We found no significant difference in average performance between the PRoPHET and GPDMI routing algorithms for the remaining battery ranges.

In *E* = [50, 100] and *E* = [75, 100] energy ranges, there are significant differences between the performance of the *e*GPDMI and the GPDMI and PRoPHET algorithms, with rates of approximately 80% and 85% delivery for the proposed algorithm, while GPDMI and PRoPHET were in the 25% range for energy cooperation in the *E* = [75, 100] range and 40% in delivery performance when *E* = [50, 100].

Finally, the Tukey test also showed differences in the performance of delivery rates between all the algorithms in the *E* = [0, 100] range. PRoPHET and GPDMI delivered approximately 85% of the messages on the network, and *e*GPDMI did not exceed 70%.

The exact values for the Tukey test are described in Table 9. These results support the previous ones in the message delivery probability, where our proposal (*e*GPDMI) achieved statistically different performance for all the battery load limits. Statistically, it is possible to compare the differences between the means.

### 6.5. Reality Scenario

**Figure 6:**
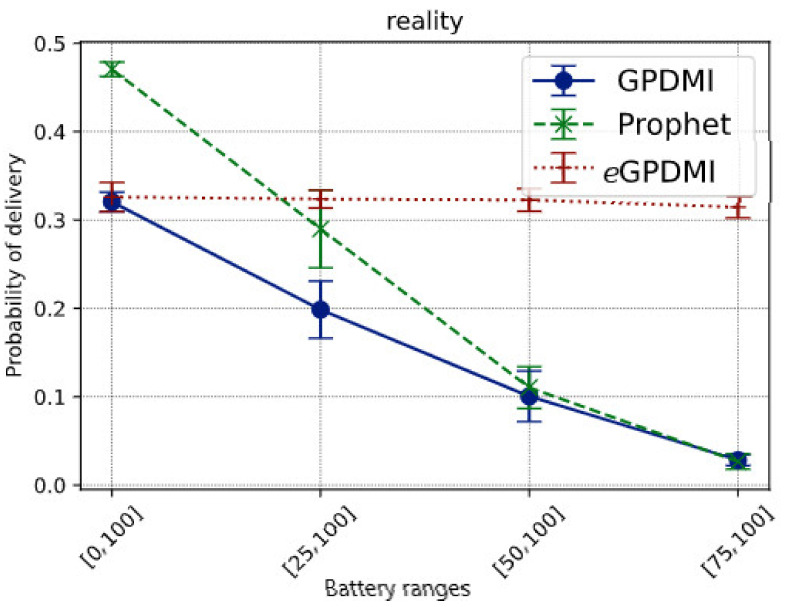
Impact of battery ranges on the delivery probability of messages in the Reality scenario.

Reality is a sparser scenario. We simulated only a few days of the entire trace, as mentioned in Table 6. Hence, it is expected that the message delivery probability would be lower. Figure 6 presents the value of the delivery probability of messages for all the tested routing algorithms.

The results show that *e*GPDMI can stay stable even when the battery range decreases. Additionally, as the battery range decreases, the delivery rate by GPDMI decreases from 32% to below 5%. PRoPHET protocol goes from 48% to less than 5%; meanwhile, *e*GPDMI goes from 32% to 30%.

**Figure 7:**
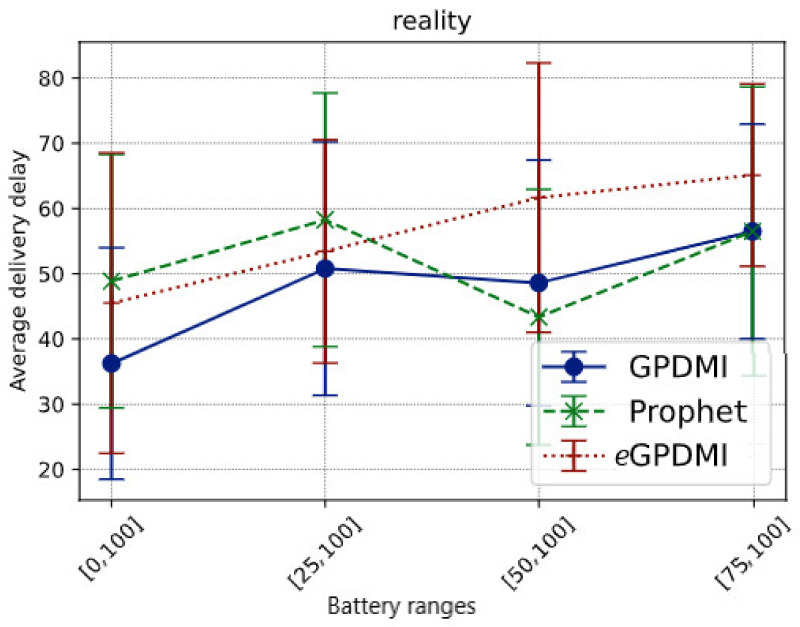
Average delivery delay vs. battery ranges in Reality scenario.

Comparing the data in Figure 6, we see that *e*GPDMI had a performance of approximately 142.85% better in the delivery probability regarding GPDMI and PRoPHET. Moreover, we remarked that *e*GPDMI had a worse performance of average delivery delay, around 13% difference (Figure 7). However, analyzing the results of the average delivery delay between *e*GPDMI and GPDMI, the standard deviation was 18.53 and 21.82, respectively. These values indicated that there was not a significant difference between the results. Thus, an unpaired *t*-test was applied to find the value of *p* = 0.3533, keeping the null hypothesis H_0_ prevailed, mean of *e*GPDMI = mean of GPDMI. It is essential to highlight that the same test was applied to *e*GPDMI and PRoPHETto obtain a *p*-value > 0.05.

It is important to emphasize that the performance of average delivery delay is statistically similar for GPDMI and PRoPHET. Furthermore, the average delivery delay was close between *e*GPDMI and the other routing algorithms in all battery ranges cases.

**Figure 8:**
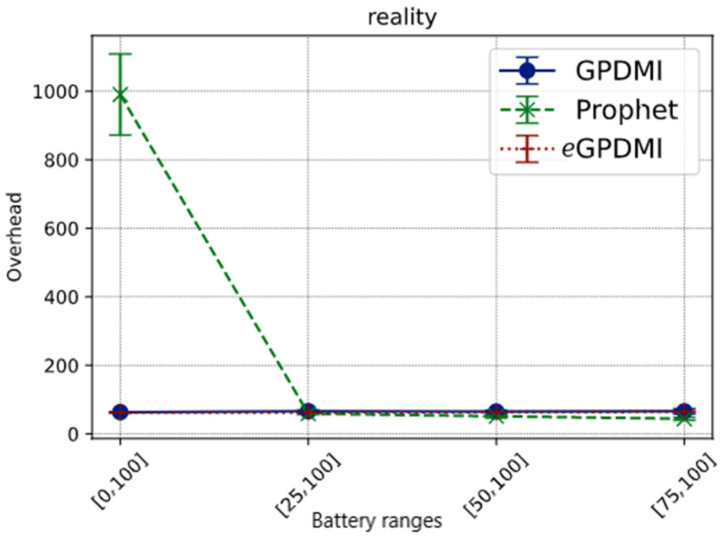
Network overhead vs. battery ranges in Reality scenario.

In Figure 8, in the cases where the minimum battery load limit for nodes to cooperate is greater than 25%, the overhead is less than 100 for all routing algorithms. On the other hand, when *E* = [0, 100], we found the overhead for the PRoPHET routing protocol to be about ten times higher than GPDMI and *e*GPDMI. The hypothesis for this result is that in PRoPHET, the forwarding probabilities take longer to adjust since Reality is a sparse scenario.

These results further demonstrate that *e*GPDMI mitigates network overhead, while stabilizing the message delivery probability in both scenarios.

### 6.6. ANOVA—Delivery Probability of Messages—Reality Scenario

The results for ANOVA in the Reality scenario are described in Table 10.

There is at least one routing algorithm with relevant performance differences in message delivery probability for the variation in the battery ranges. By doing the *F* test, using 5% of significance, we have *F* > *Fcritical*, being *F* = 414.05 for the battery range and *F* = 299.04 for the routing algorithms, values respectively higher than the Fcritical of each one.

Hence, it is reasonable to state the impact of each battery range on the delivery probability of messages. These results also support the authors’ findings in [14], which evaluated the impact of low cooperation on network performance.

Thus, such as the analysis made in the Infocom scenario, the null hypothesis H_0_ is rejected where all averages are equal. We then applied the Tukey test and statistically calculated the variations.

### 6.7. Tukey Test—Reality Scenario

Using the Tukey test, we calculated HSD = 0.032. After analyzing the impact between the message delivery probability results of the routing algorithms, we concluded that there were significant differences in the performance averages between all routing algorithms in the battery range *E* = [25, 100].

In the battery range *E* = [0, 100], there was no difference between the performance of the proposed algorithm concerning the GPDMI algorithm. However, there was a slight difference between the *e*GPDMI algorithm with 32% and the PRoPHET algorithm with 48% delivery rates.

The relationship between the PRoPHET algorithm with 48% concerning GPDMI and 32% shows that the last two comparisons maintained an average of similar deliveries.

The calculation of the absolute difference of the averages (module of the averages) supports the delivery rates in Figure 6, where *E* = [25, 100], the GPDMI algorithm had 20%, PRoPHET 30%, and *e*GPDMI 32% of the message delivery probability. In the limits of *E* = [50, 100] and *E* = [75, 100], no significant differences were found between the comparison of the PRoPHET and GPDMI algorithms.

Some differences were found in other algorithms when comparing both limits. *e*GPDMI maintained the best results compared with GPDMI and PRoPHET. The proposal (*e*GPDMI) had about 32% and 30% of the delivery performance over 10% against less than 5% of the other algorithms. In summary, *e*GPDMI obtained a statistically different performance in almost all battery ranges, except compared to GPDMI in the *E* = [0, 100] range. The exact values for the Tukey test are described in Table 11.

## 7. Conclusions and Future Works

In this paper, we presented a new routing mechanism that groups nodes inside two clusters, the cluster of highest energy level nodes and the clusters based on the profile interest of each node. We performed a questionnaire and factorial design to confirm that the energy range used for nodes to cooperate on communication has a significant negative impact on the probability of delivery of the messages.

The results showed that *e*GPDMI can mitigate the degradation of messages delivery probability when nodes are unwilling to cooperate due to their energy level. It is reasonable to assume that this happened due to the formation of groups composed of nodes with similar energy levels within a battery range considered by the nodes as high for cooperation in the network. *e*GPDMI had a general performance that was better than PRoPHET and GPDMI in higher energy level restriction scenarios, especially when the battery range for nodes cooperation was *E* = [75, 100] (only nodes with high energy level cooperated). In addition, by applying the *t*-test, no evidence of a significant difference was found among *e*GPDMI, GPDMI, and PRoPHET in the average delivery delay metric, even with a better performance of *e*GPDMI in message deliveries.

The current work has a wide range of uses in OppNets. Since there is a lack of studies that consider battery a critical parameter for routing strategy implementations, it is relevant to design protocols and other algorithms to balance energy usage, the nodes cooperation, and the messages delivery probability.

As for future work, we intend to collect accurate data on human mobility with a history of energy consumption and battery percentages of users’ mobile devices to evaluate the *e*GPDMI assessment experiments, applying machine learning techniques to learn about current energy levels of nearby devices. Besides, additional factors must be considered, such as time for recharging the battery, TTL of messages, and contacts duration.

## Figures and Tables

**Table 1:** Questionnaire about user behavior in usage of their battery of mobile devices in OppNets.

Questions	Alternatives
(i) What do you do if someone sends you a contact request on an opportunistic network while your battery level is critical?	(a) accept the request (b) do not accept the request (c) wait for the battery to recharge
(ii) What battery level would make you leave an opportunistic communication?	(a) below 10% (b) between 10% and 20% … (j) between 91% and 100%

**Table 2:** Factors of experiments.

Factor	Minimum Value (−1)	Maximum Value (1)
x1—Scenario density	Reality	Infocom
x2—Battery range	75, 100	0, 100
x3—Buffer size	10 M	100 M
x4—Message generation	600, 3600	100, 1000

**Table 3:** Variation of Xi vs. result of variable *y*.

#	x1	x2	x3	x4	*y*
1	−1	−1	−1	−1	0.0246
2	−1	−1	−1	1	0.2308
3	−1	−1	1	−1	0.4757
4	−1	−1	1	1	0.856
5	−1	1	−1	−1	0.035
6	−1	1	−1	1	0.0682
7	−1	1	1	−1	0.5025
8	−1	1	1	1	0.8496
9	1	−1	−1	−1	0.0382
10	1	−1	−1	1	0.2308
11	1	−1	1	−1	0.4641
12	1	−1	1	1	0.8596
13	1	1	−1	−1	0.0259
14	1	1	−1	1	0.2393
15	1	1	1	−1	0.4719
16	1	1	1	1	0.9029

**Table 4:** Factor evaluation.

Variation Portion	
Msg gen	0.14%
Buffer size	0.03%
Buffer size, msg gen	0.12%
**Battery range**	**76.96%**
Battery range, msg gen	0.10%
Battery range, buffer size	0.20%
Battery range, buffer size, msg gen	0.05%
**Scenario**	**18.47%**
Scenario, msg gen	0.27%
Scenario, buffer size	0.09%
Scenario, buffer size, msg gen	0.26%
**Scenario, battery range**	**3.15%**
Scenario, battery range, msg gen	0.02%
Scenario, battery range, buffer size	0.09%
Scenario, […] msg gen	0.06%

**Table 5:** Mobility dataset parameters.

	Reality	Infocom5
Device type	Phone	iMote
Duration (days)	246	3
Number of nodes	97	41
Number of meetings	*≈*54,667	*≈*22,459

**Table 6:** Simulation parameters.

Parameters	Definitions
Battery range (%)	*E* = [0, 100] *E* = [25, 100] *E* = [50, 100] *E* = [75, 100]
Routing	PRoPHET—*e*GPDMI—GPDMI
Network interface Buffer size Simulation time	Bluetooth 10 M *≈*3.1 days (274,884 s) to infocom5 *≈*10 days (864,000) to reality

**Table 7:** Classification used for the formation of energy and interest groups.

Classification	Energy	Interest
Groups	Group 1—High Energy Group 2—Low Energy	1. PowerControl 2. RoutingProtocols 3. Multicast/anycast […]
Routing	*e*GPDMI	*e*GPDMI/GPDMI

**Table 8:** Analysis of variance vs. delivery rate in scenario Infocom.

Variation	*SQ*	*gl*	*MS*	*F*	*p*-Value	*F*-Critical
Battery Range	2.62350745	3	0.874502483	295.1720982	7.154413 × 10^−52^	2.688691468
Routing Algorithm	2.426933741	2	1.213466871	409.583242	3.787614 × 10^−51^	3.080386863
Battery × Routing Algorithm	2.733700332	6	0.455616722	153.7849764	1.544337 × 10^−50^	2.183656883
Inside	0.319970176	108	0.002962687			
Total	8.104111699	119	0.874502483			

**Table 9:** Tukey test applied to the ANOVA results obtained in Table 8.

**Battery Range 25–100**	**Average 1**	**Average 2**	**Average Module**
PRoPHET × GPDMI	0.65432	0.5442	0.11012
*e*GPDMI × GPDMI	0.85301	0.5442	0.30881
*e*GPDMI × PRoPHET	0.85301	0.65432	0.19869
**Battery Range 50–100**	**Average 1**	**Average 2**	**Average Module**
PRoPHET × GPDMI	0.41614	0.398	0.01814
*e*GPDMI × GPDMI	0.85175	0.398	0.45375
*e*GPDMI × PRoPHET	0.85175	0.41614	0.43561
**Battery Range 75–100**	**Average 1**	**Average 2**	**Average Module**
PRoPHET × GPDMI	0.19725	0.15777	0.03948
*e*GPDMI × GPDMI	0.8604	0.15777	0.70261
*e*GPDMI × PRoPHET	0.8604	0.19725	0.66313
**Battery Range 0–100**	**Average 1**	**Average 2**	**Average Module**
PRoPHET × GPDMI	0.8771	0.85198	0.02512
*e*GPDMI × GPDMI	0.6783	0.85198	0.17364
*e*GPDMI × PRoPHET	0.6783	0.8771	0.19876

**Table 10:** Analysis of variance vs. delivery rate in Reality scenario.

Variation	*SQ*	*gl*	*MS*	*F*	*p*-Value	*F*-Critical
Battery Range	1.077451696	3	0.359150565	414.0508078	4.656932 × 10^−59^	2.688691468
Routing Algorithm	0.518781447	2	0.259390724	299.0415415	9.261242 × 10^−45^	3.080386863
Battery × Routing Algorithm	0.574260028	6	0.095710005	110.3403658	9.81607 × 10^−44^	2.183656883
Inside	0.093679955	108	0.000867407			
Total	0.07988524	119				

**Table 11:** Tukey test applied to the ANOVA result obtained in Table 10.

**Battery Range 25–100**	**Average 1**	**Average 2**	**Average Module**
PRoPHET × GPDMI	0.28978	0.19852	0.09126
*e*GPDMI × GPDMI	0.32365	0.19852	0.12513
*e*GPDMI × PRoPHET	0.32365	0.28978	0.03387
**Battery Range 50–100**	**Average 1**	**Average 2**	**Average Module**
PRoPHET × GPDMI	0.11035	0.10047	0.00988
*e*GPDMI × GPDMI	0.32269	0.10047	0.22222
*e*GPDMI × PRoPHET	0.32269	0.11035	0.21234
**Battery Range 75–100**	**Average 1**	**Average 2**	**Average Module**
PRoPHET × GPDMI	0.02669	0.02825	0.00156
*e*GPDMI × GPDMI	0.31454	0.02825	0.28629
*e*GPDMI ×PRoPHET	0.31454	0.02669	0.28785
**Battery Range 0–100**	**Average 1**	**Average 2**	**Average Module**
PRoPHET × GPDMI	0.47052	0.32056	0.14996
*e*GPDMI × GPDMI	0.32605	0.32056	0.00549
*e*GPDMI × PRoPHET	0.32605	0.47052	0.14447
